# Novel quantification of the regional strain distribution in the anterior cruciate ligament in response to simulated loading using micro-CT imaging

**DOI:** 10.1186/s40634-021-00416-0

**Published:** 2021-10-22

**Authors:** Alexandra M. Blokker, Ryan Wood, Jaques C. Milner, David W. Holdsworth, Timothy A. Burkhart, Alan Getgood

**Affiliations:** 1 School of Biomedical Engineering, Western University, London, ON Canada; 2 Fowler Kennedy Sports Medicine Institute, Western University, London, ON Canada; 3 Robarts Research Institute, Western University, London, ON Canada; 4 Department of Medical Biophysics, Western University, London, ON Canada; 5 Kinesiology and Physical Education, University of Toronto, 55 Harbord St, Toronto, ON M5S 2W6 Canada; 6 Fowler Kennedy Sport Medicine Clinic, London, ON Canada; 7 Department of Surgery, Western University, London, ON Canada

**Keywords:** Anterior cruciate ligament, Regional strain, Motion simulator, Medical imaging, Cadaveric specimen

## Abstract

**Purpose:**

A large percentage of anterior cruciate ligament (ACL) surgical reconstructions experience sub-optimal outcomes within 2 years. A potential factor contributing to poor outcomes is an incomplete understanding of micro-level, regional ACL biomechanics. This research aimed to demonstrate a minimally invasive method that uses micro-CT imaging to quantify regional ACL strains under clinically relevant joint loading.

**Methods:**

A pattern of 0.8 mm diameter zirconium dioxide beads were arthroscopically inserted into four regions of the ACL of four cadaveric knee specimens (mean [SD] age = 59 [9] years). A custom micro-CT compatible joint motion simulator then applied clinically relevant joint loading conditions, while an image was acquired at each condition. From the resulting images, strains within each region were calculated using the centroid coordinates of each tissue-embedded bead. Strain repeatability was assessed using the mean intra-specimen standard deviation across repeated load applications. A one-way repeated measures ANOVA (α = 0.05) was used to determine regional strain variations.

**Results:**

The mean intra-specimen standard deviation across repeated load application was ±0.003 strain for all specimens. No statistically significant differences were found between tissue regions, although medium and large effect sizes (0.095–0.450) suggest that these differences may be clinically relevant.

**Conclusions:**

The method presented here demonstrates a minimally invasive measurement of regional ACL strain under clinically relevant joint loads using micro-CT imaging. The strain measurements demonstrated excellent reliability across the five repeated load applications and suggest a non-homogenous distribution of strain through the ACL.

## Background

Despite being a highly developed and widely studied procedure, up to 10% of anterior cruciate ligament reconstruction (ACLR) cases experience acute failure within 2 years post-surgery [1, 20]. Approximately 25% of patients report persisting instability, which has been highly correlated with altered joint kinematics [20, 23]; subsequently linked to cartilage thinning [2], early onset osteoarthritis [22], reduced joint function, and loss of mobility. One potential factor contributing to sub-optimal surgical outcomes is an incomplete understanding of the micro-level biomechanics during normal physiological use, including regional tissue-strain variations in the native ACL [6]. Thus, in order to reinstate the knee joint’s native (i.e., intact) kinematics, the native ACL strain distribution should be fully characterized in high resolution under clinically relevant joint loads.

Previous approaches to quantifying intact soft tissue strain in response to joint loading fall into two categories: direct instrumentation of the tissue (e.g., differential variable reluctance transducers [DVRTs]; Hall Effect strain transducers [HESTs]) [6, 17, 19], and use of radiographic imaging to track tissue-embedded radiopaque markers (e.g., radiostereometric analysis [RSA]). Methods which directly instrument the tissue were inherently restricted to measure strain in only the lower third of the anteromedial bundle (AMB) of the ACL to prevent the surface-mounted instrumentation from impinging with surrounding structures in the joint capsule [6, 19]. While this provided useful information about the region of tissue directly instrumented, it missed potentially important regional in-homogeneities along the length of the ACL [10]. Past studies have demonstrated potential for using RSA to non-invasively determine the position of radiopaque markers embedded in soft tissue structures as they deform under load [10, 14]. However, the method has not yet been applied to the measurement of intact tissue strain in response to clinically relevant joint loads. One limitation of this method is RSA imaging resolution. The current gold standard for ligament tissue strain measurement is the DVRT, which can reportedly resolve strain differences as low as 0.0014 strain over a 5 mm region [3]. Although RSA is a high resolution (~ 0.10 mm) imaging modality [4], it cannot capture the same strain resolution as the DVRT. Micro-computed tomography (micro-CT) imaging has demonstrated higher spatial resolutions than RSA, allowing improved visualization of the joint [13, 21]. Thus, micro-CT imaging could provide similar strain measurement resolution as the current gold standard, with the potential to measure multiple regions of the tissue in a minimally-invasive manner. This method has not yet been utilized to quantify regional strain distributions in the ACL.

Therefore, the purpose of this investigation was twofold: i) to demonstrate the feasibility of using micro-CT imaging and radiopaque beads to quantify ACL strains in an intact cadaveric model in response to clinically relevant (e.g.*,* pivot shift, Lachmans) loading; and ii) to quantify the regional strain distribution along the length of the ACL and to determine if differences exists in different regions in both the axial and trans-axial directions. We hypothesized that there would be regional strain differences through the ACL in response to clinically relevant loading.

## Methods

### Specimen preparation

Four fresh-frozen human cadaveric knee joint specimens (Science Care Inc., AZ, US) (mean [SD] age: 59 [9] years, 1 male, 2 right) with no visible damage to the ACL, posterior cruciate ligament (PCL), or menisci (inspected arthroscopically prior to testing) were used in accordance with tissue use and ethical guidelines (Approval Number: MW 030217). The specimens were kept frozen at − 20 °C and thawed for approximately 18 h prior to use. All specimens were sectioned at the mid-femur and mid-tibia and 75 mm of soft tissue was removed to expose the proximal femur and distal tibia. Soft tissue surrounding the joint was left intact.

**Fig. 1:**
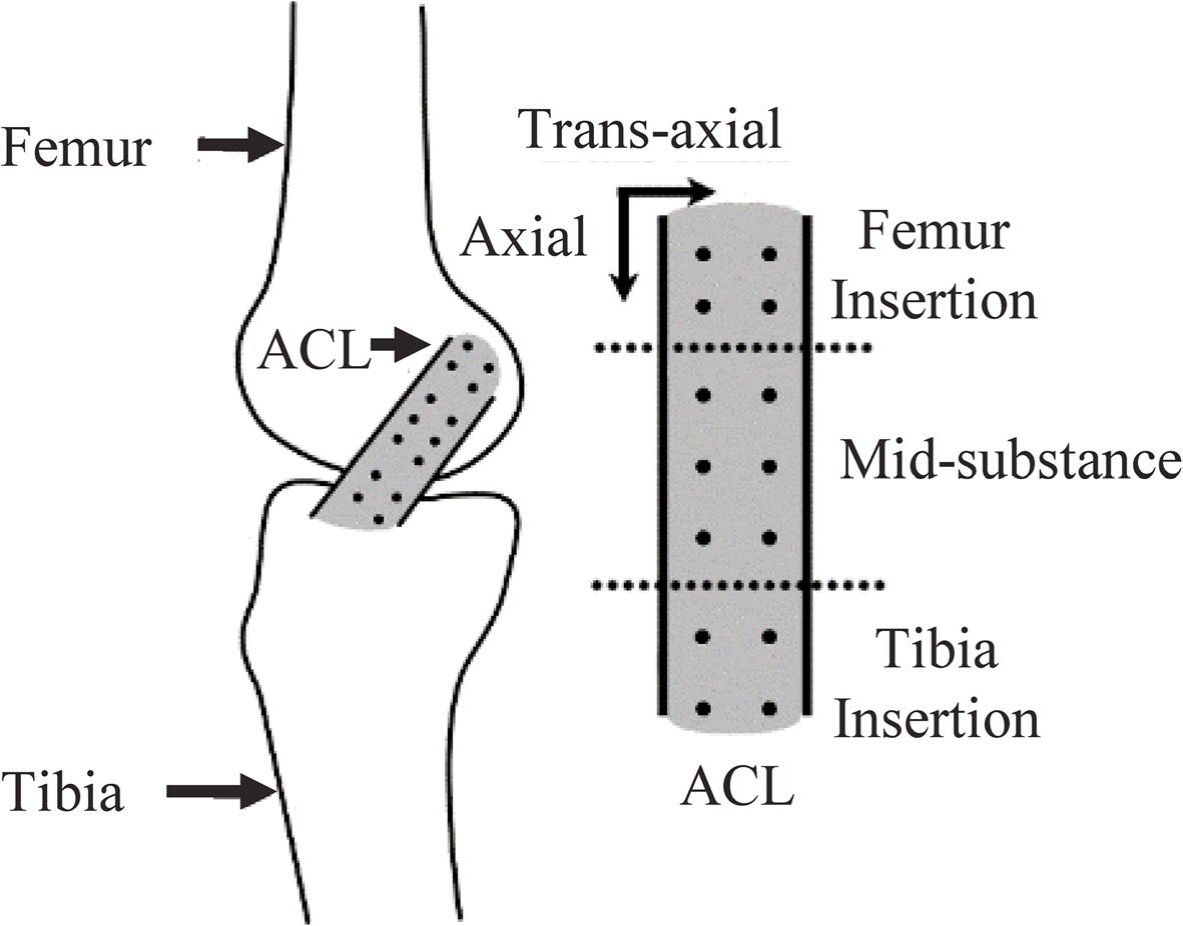
Schematic representation of the knee joint and ACL showing the approximate arrangement of beads in the ACL sub-divided into the femur insertion, mid-substance, and tibia insertion regions

With the femur secured to an arthroscopic extremity holder (Model 1650 Sawbones®, Pacific Research Laboratories, Inc., WA, US), 14 small diameter (0.8 mm diameter) zirconium dioxide (ZrO_2_) beads (.8 mm-c ZrO_2_ Ball Grade 10, Boca Bearing Inc., FL, US) were arthroscopically implanted into the anteromedial bundle (AMB) of the ACL by a fellowship-trained orthopedic surgeon. Beads were embedded into the soft tissue with an 18-gauge needle (BD Precision Glide, NJ, US) and the depth was controlled using the top of the needle bevel as a reference (~ 3 mm). The beads were embedded approximately 3–5 mm apart in two axially directed (i.e.*,* along the length of the ACL) columns spaced approximately 3–5 mm apart in the trans-axial (i.e.*,* along the width of the ACL) direction in the following regions (Fig. 1): i) four beads were embedded in the femoral footprint in a square configuration with two directly at the ligament enthesis; ii) six beads were embedded in three pairs at the mid-substance; and iii) four beads were embedded in the tibial footprint, with two at the enthesis. Once the beads were embedded, the exposed tibia and femur bone segments were potted into 75 mm and 50 mm diameter sections of ABS tubing, respectively, via dental cement (Denstone Dental Cement, Hereaus Holdings GmbH, Hanau, Germany) [7]. Previous research has shown that insertion of the beads does not affect the mechanical properties of the tissues and the beads do not move within the tissue [8].

### Experimental protocol

The potted specimens were rigidly secured within a custom-designed and validated micro-CT compatible five degree-of-freedom (DOF) knee joint motion simulator [7]. Briefly, the simulator applies closed-loop load control to the cadaveric knee while the joint is centered within the bore of a micro-CT scanner (GE Locus Ultra, London, Ontario; standard anatomical protocol: 80 kVp, 50 mA, 0.15 mm isotropic voxels; 16 s scan time); all loads were applied to the tibia while the femur remained fixed. The specimens were pre-conditioned with ten cycles of joint distraction at 0.25 Hz from − 90 to 90 N, ten cycles of internal rotation from 0 to 5 Nm at 0.25 Hz, followed by ten cycles of anterior load from 0 to 100 N at 0.25 Hz. A baseline (i.e.*,* unloaded) image of the joint was acquired with a 10 N compressive load for joint stabilization, followed by a series of loading conditions: i) 5 Nm internal rotation with a 10 N compressive load; ii) 100 N anterior translation force with a 10 N compressive load; and iii) a simulated pivot shift composed of a 100 N compression force, 5 Nm internal rotation, 10 Nm valgus rotation, and 100 N anterior translation force. Each of load condition was repeated five times at 0°, 15°, and 30° of knee flexion with an image acquired at each the load targets. A new baseline image was acquired at each new flexion angle. The scan was initiated immediately upon reaching the target force or moment and takes approximately 16 s to complete. Previous research evaluating this system suggests there is no noticeable creep in the system over this duration [7].

### Data analysis

The centroid location (x, y, z coordinate) of each bead was computed using custom developed software using a user input threshold (in HU) and user input seed points to create a cube around the surface of each bead. The centroid of the cube was then calculated in the scanner coordinate system. After the beads were located, a second, custom designed software program (MATLAB R2017b, MathWorks, MA, US; standard toolkits) was used to calculate the tissue strains. This program calculated a change in length within the ligament by comparing the Euclidean distance between adjacent beads before and after an applied load. These two inter-bead distances were then used to calculate tissue strain.

The bead pattern allowed strain to be calculated within the following regions of the ACL: i) the femoral footprint; ii) the superior mid-substance region (mid 1); iii) the inferior mid-substance region (mid 2); iv) the tibia footprint; and v) a global axial strain calculated between beads embedded in the femur and tibia footprints. The double column configuration allowed for trans-axial strains to be measured in each of these regions. A previous study that evaluated this strain calculation method found that it is accurate and precise, measuring to within 0.006 RMSE for strains as low as 0.007 strain using the same scanning and threshold parameters [9].

### Statistical analysis

Across-trial reliability was calculated using a one-way repeated measures ANOVA (with trial number as the independent variable) and intraclass correlation coefficients (ICCs) (two-way random, absolute agreement, single measures). The following ICC intervals were used to define the magnitude of reliability [11]: ICC < 0.4 = poor, 0.4 < ICC < 0.59 = fair, 0.6 < ICC < 0.74 = good, and ICC > 0.74 = excellent. Variability that can be attributed to the measurement technique was assessed by calculating the mean intra-specimen standard deviation across repeated load applications for each specimen. This was repeated for all load conditions. A set of one-way repeated measures ANOVAs were then performed to determine if the strain varied significantly across the different regions of the ACL. The ANOVAs were performed independently for the axial and trans-axial strains, and post-hoc comparisons were conducted using a Bonferroni adjustment. Effect sizes were also calculated in the form of the partial eta squared (η^2^) and evaluated according to Maher et al. [18]. All statistics were performed using SPSS (Version 25, IBM, NY, US) with α = 0.05 for all tests.

## Results

### Bead placement

**Fig. 2:**
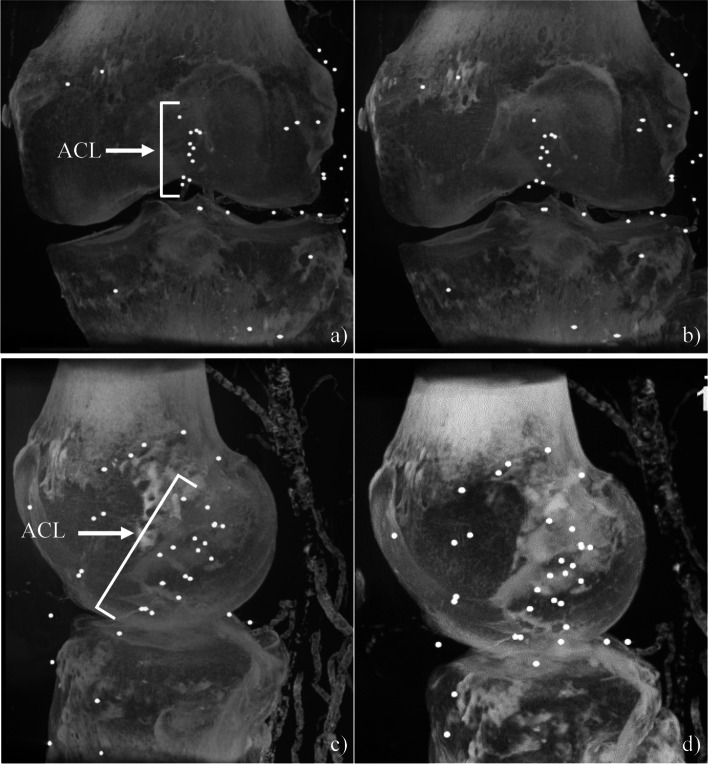
Maximum Intensity Projections (MIP) of a cadaveric specimen before [**a** coronal view, **c** sagittal view], and after [**b** coronal view, **d** sagittal view] a simulated pivot shift with loads of 100 N compression, 5 Nm internal rotation, 10 Nm valgus rotation, and 100 N anterior translation force at 0° flexion. The patella was digitally removed in post-processing in these images, for ease of viewing the joint capsule. Additional beads were also implanted into the lateral soft tissue structures but were analyzed as part of a subsequent study

All images provided sufficient contrast to threshold the radiopaque markers (ZrO_2_ > 32,767 HU) from surrounding tissues (e.g., cortical bone = 2000–3000 HU, soft tissue = 100–300 HU). The entire joint capsule was captured within the imaging FOV for all loads and flexion angles and the different regions were distinguishable (Fig. 2).

### Strain variability

One specimen was not tested in anterior translation or pivot shift loads at 30° of flexion due to interference with the imaging bore; therefore these cases were reported for the three specimens completed. No significant differences were found in the strain measured in each region across the five repeated trials for the axial strains (*p* > 0.05) with the exception of three cases: i) femur region at 30° flexion, 5 Nm IR (*p* = 0.045), ii) inferior mid-substance region at 30° flexion, pivot shift (*p* = 0.025), and iii) global strain at 30° flexion, 5 Nm IR (*p* = 0.032) (Table 1). Post hoc tests of these cases failed to locate where the significant differences occurred. Repeated axial strain measurements were found to have excellent reliability for all cases with ICCs greater than 0.799 (Table 1).

**Table 1:** ICC and *p* values calculated for axial strains measured across five repeated trials at each load condition, for each region in the AMB

Flexion Angle (°)	Load Condition	Femur Insertion		Superior Mid-Substance		Inferior Mid-Substance		Tibia Insertion		Global	
		ICC	*p* value	ICC	*p* value	ICC	*p* value	ICC	*p* value	ICC	*p* value
0	5 Nm IR	0.971	0.233	0.969	0.488	0.999	0.924	0.951	0.411	0.998	0.968
	100 N AT	0.934	0.488	0.984	0.661	0.994	0.762	0.948	0.297	0.973	0.738
	Pivot Shift	0.892	0.243	0.969	0.202	0.998	0.148	0.962	0.359	0.962	0.359
15	5 Nm IR	0.987	0.966	0.966	0.459	0.986	0.574	0.82	0.63	0.920	0.448
	100 N AT	0.999	0.115	0.993	0.061	0.999	0.888	0.835	0.394	1.000	0.799
	Pivot Shift	0.999	0.71	0.99	0.685	0.998	0.148	0.985	0.438	1.000	0.124
30	5 Nm IR	0.99	0.045*	0.964	0.171	0.994	0.902	0.919	0.689	0.997	0.032*
	100 N AT	0.991^a^	0.434	0.799^a^	0.112	0.999^a^	0.981	0.94^a^	0.419	0.993^a^	0.216
	Pivot Shift	0.915^a^	0.981	0.964^a^	0.172	0.96^a^	0.025*	0.974^a^	0.555	0.999^a^	0.106

*IR* Internal Rotation, *AT* Anterior Translation Force

* Post-hoc analysis failed to find significant difference

^**a**^ case with *n* = 3

No significant differences were found across repeated trials for the trans-axial strains with the exception of four regions: i) inferior mid-substance region at 0° flexion angle, 5 Nm IR (*p* = 0.006), ii) inferior mid-substance under 15° flexion angle, pivot shift (*p* = 0.016), iii) the tibia region at 30° flexion angle, 5 Nm IR (*p* = 0.037), and iv) inferior mid-substance region under 30° flexion angle, 100 N AT (*p* = 0.009) (Table 2). However, post-hoc tests failed to find significance for any of these cases. Repeated trans-axial strain measurements showed excellent reliability in 96% of cases, fair reliability in 2% of the cases (one case: 15° flexion angle, 5 Nm IR, in the femur region), and a negative ICC value in the remaining 2% of cases (one case: 30° flexion angle, 100 N AT in the femur region) (ICC = − 0.05) (Table 2).

**Table 2:** ICC and p values calculated for trans-axial strains measured across five repeated trials at each load condition, for each region in the ACL

Flexion Angle (°)	Load Condition	Femur Insertion		Superior Mid-Substance		Inferior Mid-Substance		Tibia Insertion	
		ICC	*p* value	ICC	*p* value	ICC	*p* value	ICC	*p* value
0	5 Nm IR	0.996	0.711	0.998	0.006*	0.988	0.553	0.969	0.599
	100 N AT	0.984	0.174	0.964	0.905	0.964	0.629	0.995	0.466
	Pivot Shift	0.955	0.234	0.993	0.344	0.999	0.800	0.993	0.089
15	5 Nm IR	0.524	0.438	0.985	0.952	0.991	0.985	0.994	0.953
	100 N AT	0.999	0.921	0.991	0.055	0.999	0.832	0.984	0.978
	Pivot Shift	0.999	0.541	0.989	0.617	0.999	0.016	0.984	0.978
30	5 Nm IR	0.993	0.445	0.999	0.964	0.988	0.882	0.981	0.037*
	100 N AT	−0.05^a^	0.583	0.999^a^	0.417	0.996^a^	0.009*	0.966^a^	0.951
	Pivot Shift	0.984^a^	0.694	0.971^a^	0.383	0.987^a^	0.183	0.998^a^	0.302

*IR* Internal Rotation, *AT* Anterior Translation Force

* Post-hoc analysis failed to find significant difference

^a^ Case with *n* = 3

The mean intra-specimen standard deviation across repeated load application was 0.003 strain in the axial direction for all specimens, across all load conditions (Table 3). The largest variability in the strains occurred at the femur insertion and superior mid-substance regions (mean SD = 0.004 strain), with the lowest variability in the tibia insertion region (mean SD = 0.002 strain). The intra-specimen variability was relatively consistent for each specimen (Table 3).

**Table 3:** The mean of the standard deviations for the axial and trans-axial strains measured across repeated trials for each region of the ligament, for each specimen. This represents a mean of all load conditions tested for each specimen

Specimen	Femur Insertion (strain)	Superior Mid-Substance (strain)	Inferior Mid-Substance (strain)	Tibia Insertion (strain)
Axial				
1	0.001	0.002	0.003	0.004
2	0.009	0.007	0.003	0.001
3	0.002	0.003	0.004	0.002
4	0.005	0.005	0.002	0.001
Trans-axial				
1	0.002	0.010	0.003	0.004
2	0.009	0.013	0.010	0.008
3	0.004	0.009	0.017	0.002
4	0.002	0.002	0.004	0.009

The mean intra-specimen standard deviation across repeated load application was 0.006 strain in the trans-axial direction for all specimens, across all load conditions. The largest variability in the trans-axial strains occurred at the superior mid-substance (mean SD = 0.008 strain), with the lowest variability in the femur insertion region (mean SD = 0.005 strain). The intra-specimen variability was relatively consistent for each specimen (Table 3).

### Regional variation in axial strain

**Fig. 3:**
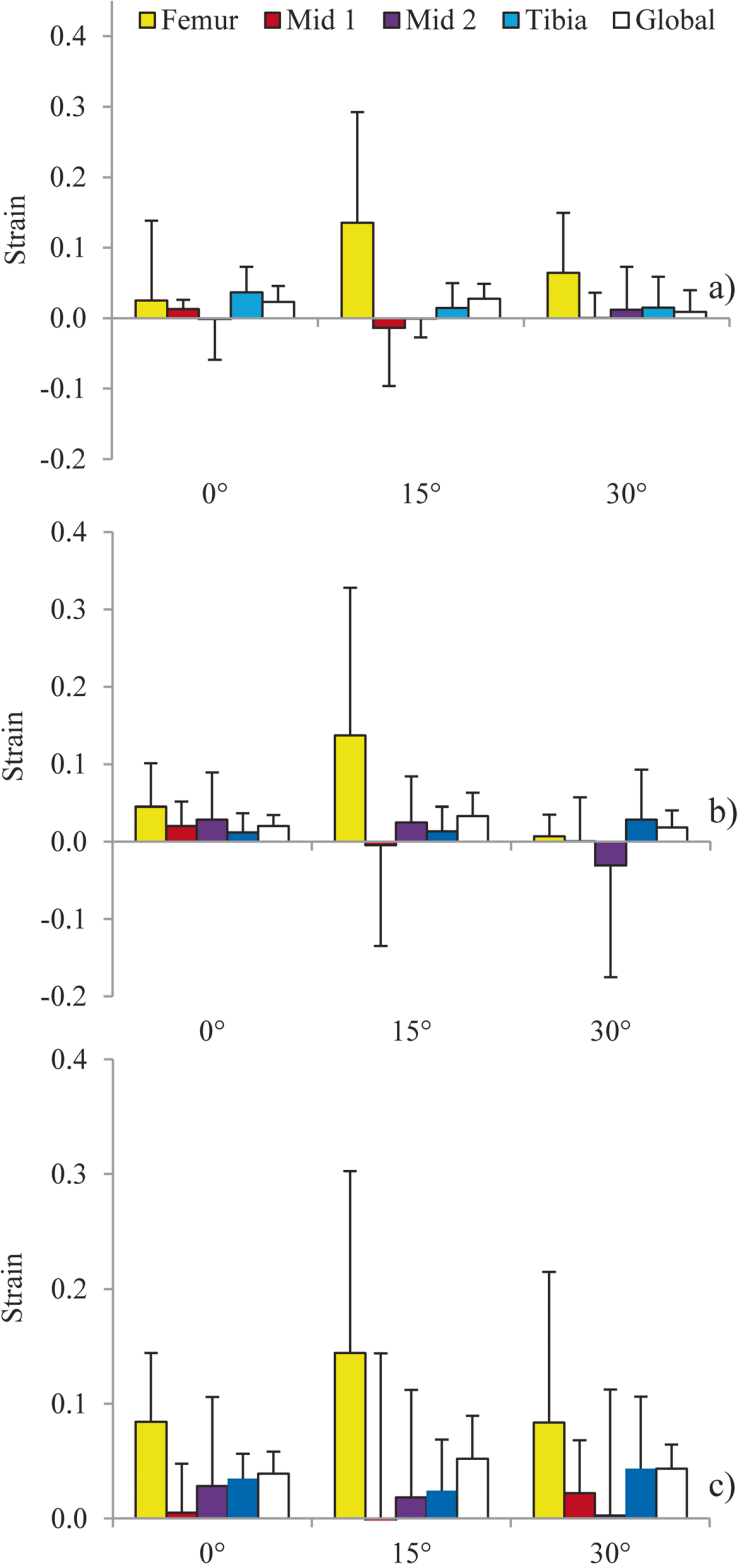
Comparison of the mean (SD) magnitude of axial strain between ACL regions and across knee flexion angles when exposed to: **a** 5 Nm internal rotation; **b** 100 N anterior translation; and **c** simulated pivot shift

No statistically significant differences were found between regional axial strains when the knee was exposed to a 5 Nm internal rotation load (*p* > 0.05) (Fig. 3a), a 100 N anterior translation load (*p* > 0.05) (Fig. 3b), or a simulated pivot shift (*p* > 0.05) (Fig. 3c) at any of the flexion angles tested. However, medium and large effect sizes were found for all regions and degrees of flexion (η^2^ = 0.107–0.448) (Table 4).

**Table 4:** Effect sizes for each of the loading conditions across the three knee flexion angles tested

	Flexion Angle		
Loading Condition	0°	15°	30°
Axial			
5 Nm IR	0.188	0.566	0.416
100 N AT	0.166	0.104	0.255
Pivot Shift	0.326	0.200	0.451
Trans-axial			
5 Nm IR	0.145	0.631	0.376
100 N AT	0.130	0.095	0.127
Pivot Shift	0.139	0.153	0.250

### Regional variation in trans-axial strain

**Fig. 4:**
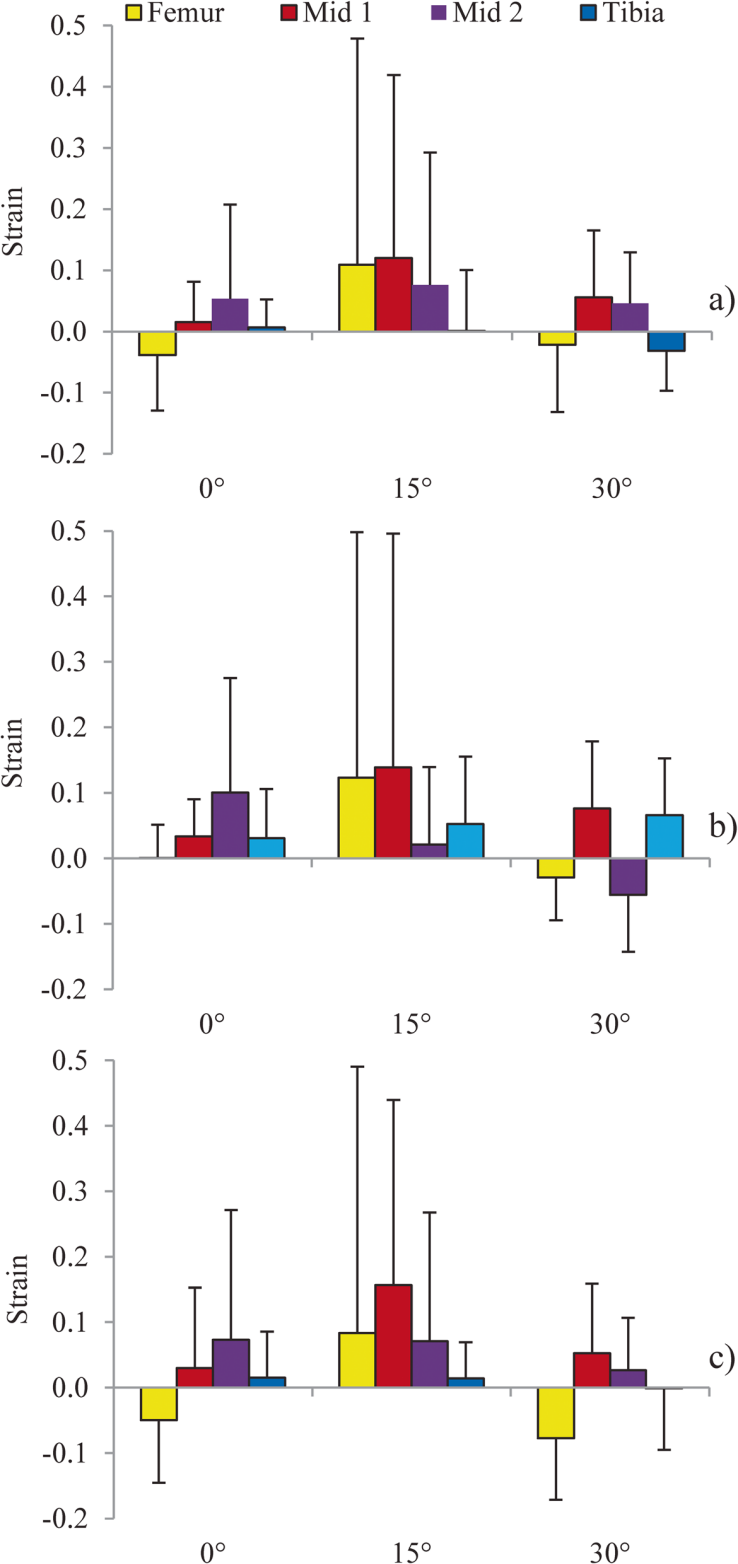
Comparison of the mean (SD) magnitude of trans-axial strain between ACL regions and across knee flexion angles when exposed to: **a** 5 Nm internal rotation; **b** 100 N anterior translation; and **c** simulated pivot shift

Similarly, no statistically significant regional differences were found between regional trans-axial strains when the knee was exposed to a 5 Nm internal rotation load (*p* > 0.05) (Fig. 4a), a 100 N anterior translation load (*p* > 0.05) (Fig. 4b), or the simulated pivot shift (*p* > 0.05) (Fig. 4c) for any flexion angle tested. However, similar to the axial strains, medium and large effect sizes were found for all loading conditions and at all flexion angles (η^2^ = 0.095–0.376) (Table 4).

## Discussion

To the author’s knowledge this is the first study to quantify regional ACL strain in response to clinically relevant joint loads (e.g.*,* pivot shift and Lachmans) using a minimally invasive micro-CT imaging-based method. The strains measured under various joint loading conditions demonstrated excellent reliability across repeated load applications, indicating that the method is highly consistent in both strain measurement and load application. No statistically significant differences were found across the five regions of the AMB in either the axial or trans-axial directions; however, effect sizes suggest potential clinically meaningful regional strain variability.

The minimally-invasive strain measurement method described here was highly repeatable, as indicated by low standard deviations in strain measured across the repeated load application trials. It was found that within a set of repeated load applications, the measured inter-bead strains varied on average by only 0.003 strain between trials. The method’s high repeatability is further supported by a lack of statistically significant differences and high ICC values in the strains measured under repeated load application. The totality of this data indicates that the majority of the variation in strain is likely attributable to inherent biological variability (across and within specimens) as opposed to the measurement technique.

The selection of a micro-CT imaging system allows the imaging of both the bone and soft tissue structures within the joint capsule envelopment of an intact tissue specimen. This is advantageous as it ensures the simultaneous measurement of both tissue strain and joint kinematics in high resolution, thus eliminating the need to register independent kinematic tracking and strain measurement systems. The error associated with 2D to 3D registration has been shown to be approximately 1.31 mm for in-plane and 2.40 mm for out-of-plane motion [15], while fiducial and surface based registration methods can produce errors on the order of 1.25 and 0.25 mm respectively [16]. These errors are essentially eliminated with the method presented here.

The current investigation demonstrated that this method is capable of locating small diameter ZrO_2_ beads embedded in soft tissue to quantify regional tissue strains. The resulting images provided a clear distinction between the bone tissue and beads without motion or material artifacts. No statistically significant differences in the distribution of strain were found throughout the AMB in either the axial or trans-axial directions. However, medium to large effect sizes found for all conditions suggest that regional strain variation may be clinical relevant and require further investigation. Butler et al. [10] also demonstrated no statistically significant variation in strain distribution along the long axis of the ACL when a cadaveric human AMB was dissected into five separate fiber bundles while maintaining the femur and tibia footprints. Butler et al. [10] showed a symmetric strain distribution along the longitudinal axis (i.e., from femur insertion to tibia insertion) of each subunit, with the two footprints demonstrating greater, non-significant, strain compared to the mid-substance (0.0025 vs. 0.01 strain, respectively). In the present study, a similar trend of higher strains in the footprints was observed when the joint was at 15° of flexion under a 5 Nm internal rotation load with non-significant difference between the footprints and mid-substance (approximately 0.02 to 0.07 strain). A similar observation was made by Hirokawa et al. [12], who proposed that increased tension at the footprints was a symptom of the un-natural insertion configuration experienced when the ligament undergoes uni-axial tension as opposed to a true in-homogeneity; however, this study was conducted with sub-units from only a single cadaveric specimen. This differs drastically from the approach presented in the present study, where the native ACL and surrounding joint structures were kept fully intact and the ligament may have been strained in more clinically and physiologically accurate directions through actuation of the femur and tibia.

The magnitude of strain measured from the methodology presented here agrees with previously reported values [5]. Berns et al. [5], instrumented cadaveric AMBs with liquid-mercury strain gauges and applied a range of passive joint motions and found, for example, a mean (SD) strain of 0.037 (0.018) under a pure 100 N anterior load at a 30° flexion; this agrees with the current method (0.035 [0.010)] strain) under the same load and flexion angle. Berns et al. [5] also demonstrated a mean (SD) strain of 0.049 (0.014) under a combined load of 100 N anterior load and 20 Nm of valgus torque, which is similar to the current study’s finding of 0.043 (0.010) in response to the simulated pivot shift. However, different strain responses were found under a 5 Nm internal rotation load at 30° flexion between the two methods, with Berns et al. [5] finding a mean (SD) strain of 0.014 (0.012) and the present study finding a mean (SD) strain of − 0.008 (0.027). One possible explanation for this difference is that Berns et al. [5] used a high stiffness sheath around the strain gauge to prevent impingement of the gauge against surrounding structures in the joint and observed that the sheath itself may have impinged with the joint during this motion; highlighting the benefit of a minimally invasive strain measurement method. Furthermore, the variability in the strain measurements between specimens found with the current method was comparable to the variability found by Berns et al. [5], indicating that despite the small sample size in the present study (*n* = 4 vs *n* = 13 by Berns et al. [5]). The strains measured can be considered relatively representative of a larger sample.

An interesting finding from this work was a compressive strain demonstrated at a 30° flexion angle when the joint was subjected to pure internal rotation and anterior loads. However, while the directionality of these strains (negative) is traditionally interpreted as compression, it is more likely, given the type and location of the tissue being tested, that this is indicative of the tissue folding onto itself and not that it is being compressed. A recent study by Hirokawa et al. [12] analyzing strain inhomogeneities using a photoelastic coating to detect fringes in the tissue found regional compressive strain at the tibial insertion with a corresponding tension in the femoral insertion at low joint flexion angles [12]. It was suggested that this was a result of the ligament having no compressive strength and therefore collapsing or folding over itself when the ligament is unloaded, which occurs at specific flexion angles [12]. In the study presented here, tissue relaxation appeared to occur through multiple regions in the AMB, including the superior mid-substance, tibia insertion, and global strain measurements.

There are some limitations that exist with the present study. First, this study was performed on a small sample of cadaveric specimens, and likely contributed to the statistical tests not finding significant differences between the regions. Second, only the AMB was instrumented as result of the small working space afforded by the arthroscopic bead insertion technique. Another limitation of this study was the difficulty of reproducibly placing the radiopaque markers in the same anatomical positions in different specimens. The surgeon implanting the markers visualized the anterior surface of the ligament with an arthroscope and, while this was the optimal view to insert the majority of the markers, the femoral insertion was difficult to view from this angle. As a result, some markers were inserted without direct line of sight. Finally, the method, as presented here, is limited to investigating joint flexion angles from 0 to 30° due to limited physical space within this specific micro-CT scanner. Despite this limitation, many physiologically important joint activities and a clinically important assessment (i.e., the pivot shift) occur in this flexion range. In addition, this dynamic joint motion simulator system is highly portable and is compatible with larger bore diameter CT-scanners.

## Conclusions

Overall, the use of embedded radiopaque markers, micro-CT imaging, and a micro-CT compatible dynamic knee joint motion simulator provides a novel and highly reliable method for measuring regional ligament strain at clinically relevant loading conditions. The use of this minimally-invasive strain measurement technique at joint loads that are seen during regular joint use provides the opportunity to fully characterize intact ACL strain across various regions in the tissue at valid strain magnitudes, or to investigate the regional properties of novel and existing graft tissues designed to replace the ACL during reconstruction.

## Data Availability

The datasets used and/or analysed during the current study are available from the corresponding author on reasonable request.
